# Prognosis of Methanol Poisoning in a Developing Setting

**DOI:** 10.34172/aim.2024.20

**Published:** 2024-03-01

**Authors:** Mohammad Reza Sasani, Hossein Molavi Vardanjani, Zahra Mehdipour Namdar, Marjan Jeddi, Shiva Seif, Sogol Sedighi, Seyed Amirreza Akhlagh, Dena Firouzabadi, Amirreza Dehghanian

**Affiliations:** ^1^Medical Imaging Research Center, Department of Radiology, School of Medicine, Shiraz University of Medical Sciences, Shiraz, Iran; ^2^Department of MPH, School of Medicine, Shiraz University of Medical Sciences, Shiraz, Iran; ^3^Student Research Committee, Shiraz University of Medical Sciences, Shiraz, Iran; ^4^Endocrinology and Metabolism Research Center, School of Medicine, Shiraz University of Medical Sciences, Shiraz, Iran; ^5^Department of Radiology, School of Medicine, Shiraz University of Medical Sciences, Shiraz, Iran; ^6^Department of Clinical Pharmacy, School of Pharmacy, Shiraz University of Medical Sciences, Shiraz, Iran; ^7^Trauma Research Center, Molecular Pathology and Cytogenetics Section, Department of Pathology, School of Medicine, Shiraz University of Medical Sciences, Shiraz, Iran

**Keywords:** Alcoholic intoxication, Brain imaging, Mortality rate, Poisoning, Prognosis, Spiral CT, Wood alcohol

## Abstract

**Background:**

Methanol-poisoning can be a challenging cause of mortality. Identifying the epidemiological, clinical, and para-clinical determinants of outcome in methanol-poisoning patients could be a step forward to its management.

**Methods:**

In this hospital-based cohort study, 123 methanol-poisoning patients were included. Data on background variables, details of methanol consumption, and laboratory assessments were recorded for each patient. Patients underwent brain CT scans without contrast. We evaluated the association of all gathered clinical and para-clinical data with patients’ outcome and length of hospital stay (LOS). Independent association of potential determinants of death, and LOS were modeled applying multivariable logistic, and Ordinary Least Square regressions, respectively. Odds ratio (OR), and regression coefficient (RC), and their 95% confidence intervals (CIs) were estimated.

**Results:**

Most of the study population were male (n=107/123). The mean age of the participants was 30.3±9.1 years. Ninety patients (73.2%) were reported as being conscious on admission, and 34.3% of patients were identified with at least one abnormality in their CT scan. Level of consciousness (LOC) (OR: 42.2; 95% CI: 2.35-756.50), and blood pH (OR: 0.37; 95% CI: 0.22-0.65) were associated with death. Supratentorial edema (RC: 17.55; 95% CI: 16.95-18.16) were associated with LOS.

**Conclusion:**

Besides LOC, patients with any abnormality in their brain CT scan on admission were found to be at higher risk of death, and patients with supratentorial edema were at risk of longer LOS. Brain CT-scan on admission should be considered as a part of the routine procedure during the management of methanol-poisoning.

## Introduction

 A massive accidental methanol-poisoning outbreak occurred in Iran in the first months of the COVID-19 epidemic in February 2019.^1^ The misleading information that supported alcohol consumption for its protective properties against COVID-19 infection and also availability of illegal alcoholic products on the black market^2^ were considered as the two most important causes of this outbreak.

 As methanol poisoning occurs, several clinical presentations may be expected. Early manifestations include non-specific symptoms such as nausea, vomiting, headache, and abdominal pain which are seen within 12-24 hours after consumption. Visual symptoms such as blurred vision appear as soon as acidosis develops. As acidosis progresses, there may be central nervous system (CNS) manifestations including memory loss, agitation, and in severe cases stupor, and coma.^3^

 Methanol is highly toxic for nerves and blood vessels and after absorption, is oxidized by alcohol dehydrogenase to formaldehyde, which is rapidly converted to formic acid. Mitochondrial cytochrome C oxidase inhibition by formic acid results in the suppression of oxidative phosphorylation and destruction of oxygen utilization in neurons. This hypoxia and necrosis can lead to neuronal degeneration and CNS dysfunction. In addition, formicacid can cause secondary edema, ischemia, hemorrhage, impairment of the blood-brain barrier, axonal demyelination, and cell death.^4^

 This pathologic CNS dysfunction has some manifestations on brain imaging, including bilateral necrosis of the basal ganglia, mostly the putamen, with or without hemorrhage, and hemorrhagic lesions in subcortical white matter on computed tomography (CT) and necrotic lesions within the globus pallidus, nucleus caudate, thalamus, cerebellum, brainstem, pons and cerebral cortex, and optic nerve atrophy on magnetic resonance imaging.^5^

 Delayed admission to the hospital and delayed diagnosis are among the factors for poor prognosis.^6,7^ Respiratory arrest on admission, high blood sugar (BS), severity of metabolic acidosis, state of consciousness, and serum ethanol levels on admission^7-9^ are also determinants. Radiologic findings such as putamen hemorrhage and insular subcortical white matter necrosis have been associated with poorer outcomes.^10^

 Most of the available evidence on the prognosis of methanol-poisoning is from developed regions, where patients may be more literate, and have different health-seeking behaviors compared with patients from developing regions such as Iran. In the developed countries’ settings, more timely and appropriate diagnostic methods may be available. Accordingly, clinical circumstance and determinants of its outcome may be different from developing settings.

 Therefore, due to lack of sufficient published data regarding management and outcome of methanol poisoning in developing countries, in this study, we aim to investigate the epidemiological, clinical, and para-clinical determinants of the outcomes of accidental methanol-poisoning in a cohort of patients in southern Iran.

## Materials and Methods

 In this hospital-based retrospective cohort study, we included 123 methanol-poisoning patients admitted to the referral hospital for poisoning management in Shiraz, Iran. The hospital was affiliated with Shiraz University of Medical Sciences. We enrolled patients with a confirmed bedside diagnosis of methanol-poisoning that were aged 18 years or above.

 The patients were enrolled in this study through convenient sampling and all admitted patients were included except those who did not consent to participate in the study. Written informed consent was taken from all the participants.

 Diagnosis of methanol poisoning was based on patient-reported history of alcohol ingestion, and clinical and paraclinical findings. Patients were followed until discharge from the hospital or in-hospital death. The primary and secondary outcomes were considered to be in-hospital death, and length of stay in the hospital (LOS), respectively. LOS was defined as the time period between time of admission up to the time of discharge. Cases who died in hospital were excluded during modeling for prediction of LOS.

 Based on a comprehensive literature review and group discussions, a conceptual framework was developed for the study, and accordingly, a data collection form was designed. Data was collected by face-to-face interviews with the patient or her/his caregiver, and medical records were reviewed. Data on the time elapsed between ingestion and admission, patient’s age and gender, patient’s pulse rate (n), systolic (SBP) and diastolic blood pressure (mm Hg), temperature (°C), O_2_ saturation (%), nausea, vomiting, blurred vision, headache, and vertigo, and laboratory factors including complete blood gas, BS (mg/dL), calcium (mg/dL), phosphate (mg/dL), aspartate aminotransferase (IU/L), alanine aminotransferase (IU/L), lactate dehydrogenase (mg/dL), creatine phosphokinase (IU/L), and levels of blood pH, HCO_3_ (mEq/L), and PCO_2_ (mm Hg) at the time of admission were recorded.

 Immediately after clinical stability of the patients during their hospital course, they underwent brain CT scan without contrast, and a board-certified radiologist with no information about the clinical data of the patient interpreted the images. CT scans were carefully assessed for identifying any lesion and its characteristics including anatomical location and unilateral or bilateral involvement.

 Treatment options including alkalization, ethanol prescription, and hemodialysis were also documented based on the patients’ charts.

 Data were cleaned and prepared for the appropriate statistical techniques.^11^ Descriptive statistics including mean, standard deviation (SD), and relative frequency (%) were used for data description. Chi-square test, Fisher’s exact test, two independent sample t-test, or Mann–Whitney U test were applied to analyze the univariate association of independent variables with outcome. Multivariable modeling of predictors of death and LOS was done using binary logistic regression and Robust multiple regression, respectively. Because of the clinical importance of different independent variables including CT findings, and LOC and also due to the higher level of multicollinearity between them, three different models were fitted for the death outcome: (1) Ignoring CT findings and level of consciousness (LOC), (2) Including CT findings and LOC, and (3) Including the LOC without CT findings. Moreover, for the LOS outcome, two different models were fitted: (1) Including the LOC, ignoring CT findings, and 2) Including CT findings, ignoring the LOC. The criterion for variable selection was a univariate *P *value less than 0.3. The backward elimination technique was applied for multivariable modeling. *P* value < 0.05 was considered statistically significant. Data were analyzed using Stata 11.2 (Stata Corporation, College Station, TX, USA).

## Results

 A total of 123 patients with methanol poisoning, including 107 male patients and 16 female patients, were analyzed. The mean age of participants was 30.3 ± 9.1 years. Ninety-four patients (76.4% of all the patients) were admitted to an internal medicine ward, while 29 patients (23.6%) were admitted to an intensive care unit (ICU).

 On arrival, after stabilization, treatment was started with hydration and alkalization with sodium bicarbonate for all patients. Ethanol treatment was prescribed for 108 patients (100 cc of 96% ethanol in 400 cc DW 5% resulting in 20% solution, prescribed based on patients’ weight, and the maintenance dose was adjusted during hemodialysis); 88 (81.5%) of these patients survived. For the intention of managing high anion gap metabolic acidosis and end-organ damage (visual changes or renal failure), 77 patients were treated with hemodialysis for rapid removal of the toxic acid and its metabolites leading to 87% (67 cases) survival rate.

###  Clinical and Biochemical Findings

 The estimated mean time from methanol consumption to admission at the hospital was 1.5 ± 1.0 days (0-4.08). On admission, gastrointestinal symptoms including nausea and vomiting were observed in 81 (66%) and 76 (62%) patients, respectively. Blurred vision was reported among 80 (65%) patients. Ninety patients (73%) were reported as being conscious on admission, while 22 (18%) and 11 (8.9%) were identified as being drowsy and comatose, respectively. The mean serum pH level at the time of admission was 7.12 ± 0.186 (6.58-7.50).

Tables 1 and 2 present the characteristics of the study participants including their symptoms, vital signs, and laboratory findings on admission in the total study population, the deceased and the surviving individuals, respectively.

**Table 1:** Characteristics of the Study Participants

**Complaint**	**Total ** **No. (%)**	**Survived ** **No. (%)**	**Dead ** **No. (%)**	***P*** ** Value**
Number of days from consumption				
≤ 2	46 (38)	41 (89.1)	5 (10.9)	0.62
> 2	75 (62)	60 (80)	15 (20)	
Nausea	81 (65.8)	73 (90.1)	8 (9.9)	0.008
Vomiting	76 (61.8)	67(88.2)	9 (11.8)	0.077
Blurred vision	80 (65.0)	72 (91.1)	7 (8.9)	0.005
Headache	25 (20.0)	24 (96)	1 (4)	0.073
Vertigo	24 (19.5)	23 (95.8)	1 (4.2)	0.119
LOC				
Conscious	90 (73.2)	89 (98.9)	1 (1.1)	0.00
Drowsy	22 (17.9)	13 59.1)	9 (40.9)	
Coma	11 (8.9)	1 (9.1)	10 (90.9)	
Acidosis				
Yes	79	59	20	< 0.001
No	44	44	0	

Assumptions of T test and chi-square were checked, and all of them were valid.

**Table 2:** Mean Vital Signs and Biochemical Findings in Study Subjects, by Survival Status

**Parameter**	**Survived (SD)**	**Dead (SD)**	***P*** Value^*^
O2 sat (%)	92.9 (6.2)	86.9 (9.3)	0.046
Pulse (n)	87.7 (11.3)	87.3 (31.3)	0.416
SBP (mm Hg)	121.7 (12.9)	98.9 (27.1)	0.00
DBP (mm Hg)	77.0 (7.4)	71.7 (10.4)	0.069
Temperature (C)	36.6 (0.4)	36.7 (0.3)	0.43
BS (mg/dL)	107.4 (35.9)	252.9 (140.2)	< 0.001
PH	7.2 (0.1)	6.8 (0.1)	< 0.001
HCO3 (mEq/L)	11.4 (6.9)	5.3 (2.7)	< 0.001
PCO2 (mm Hg)	27.6 (11.5)	32.5 (14.7)	0.12
CPK (IU/L)	288.6 (1183.5)	2105.8 (6676.4)	0.499
LDH (mg/dL)	440.8 (252.9)	473.4 (217.1)	0.32
AST (IU/L)	43.3 (53.8)	68 (86.8)	0.082
ALT (IU/L)	50.5 (55.9)	52.7 (30.1)	0.172
Calcium (mg/dL)	9.2 (0.6)	8.9 (0.8)	0.096
Phosphate (mg/dL)	13.5 (53.1)	6.6 (2.9)	0.003

SBP, Systolic blood pressure; DBP, Diastolic blood pressure; CPK, Creatine phosphokinase; LDH, Lactate dehydrogenase; AST, Aspartate aminotransferase; ALT, Alanine transaminase; ^*^P value estimated applying Mann–Whitney U test.

###  Radiological Findings

**Figure 1:**
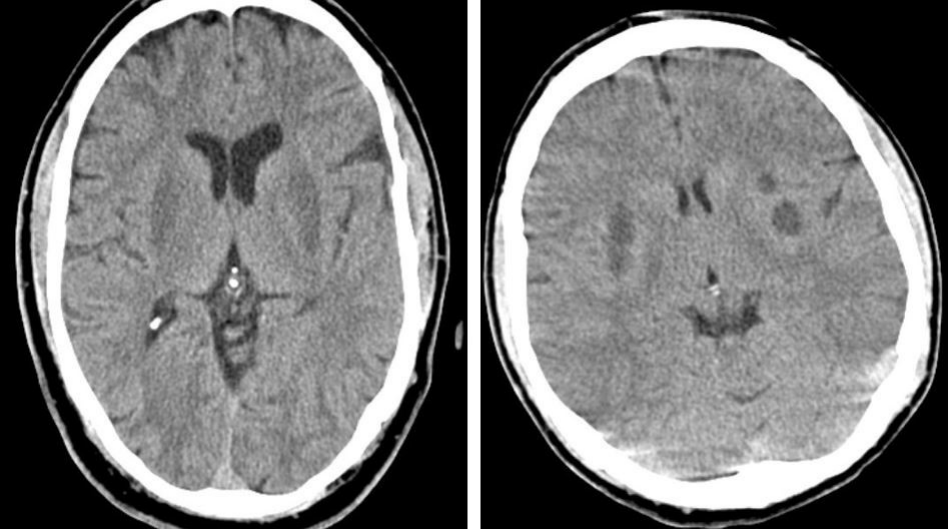
Axial Brain CT Images of Two Patients Showing Areas of Hypodensity in Both Putamens Representing Bilateral Putaminal Ischemia

**Figure 2:**
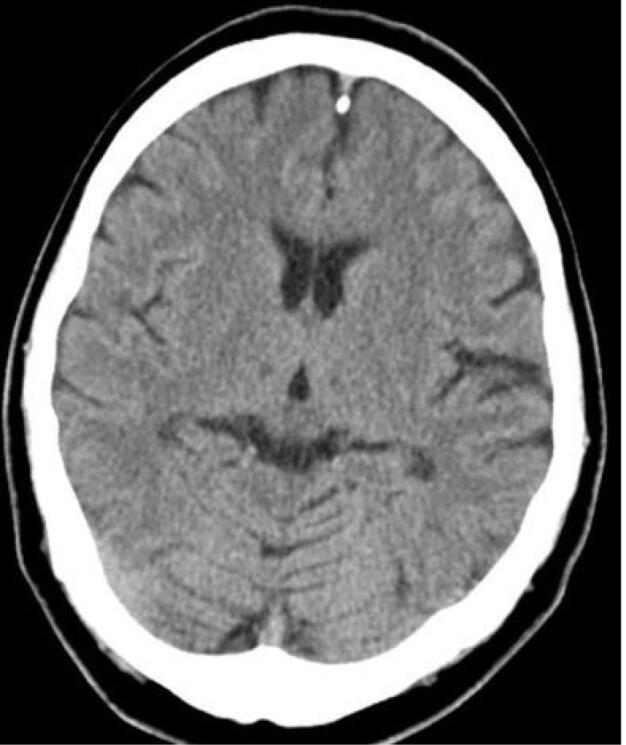
Axial Brain CT Image Showing Small Foci of Hypodensity in Both Thalami, Suggesting Bilateral Thalamic Ischemia and Necrosis

**Figure 3:**
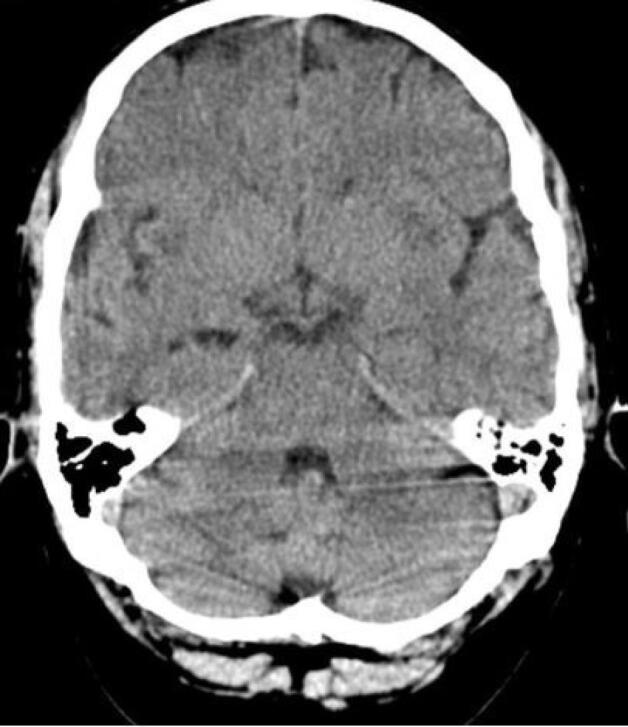
Axial Brain CT Showing Irregular Areas of Hypodensity With Small Spots of Fluid Density in the Subcortical White Matter of Insula Bilaterally, Representing Bilateral Insular Subcortical White Matter Ischemia and Necrosis.

**Figure 4:**
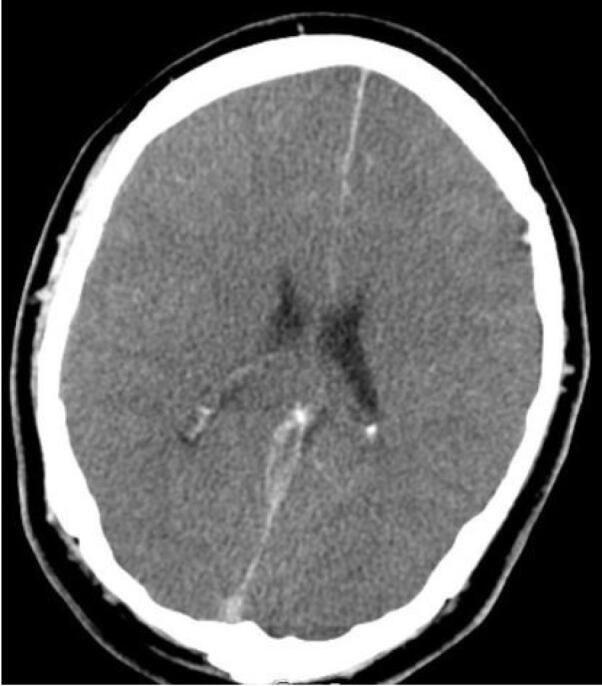
Axial Brain CT Showing the Diffuse Decreased Density of Brain Parenchyma Associated With Sulci Effacement Representing Diffuse Brain Edema

 Ninety-nine patients out of 123 underwent brain CT scans; in 34.3% (n = 34), at least one abnormality was identified in the CT scan. Twenty-one lesions (ischemia/necrosis) were detected within the putamen (Figure 1), three in the thalamus (Figure 2), two within the external capsule, and 15 in the white matter of which 4 were in the subcortical white matter of the insula (Figure 3). Moreover, 7 patients had generalized supra- and infratentorial edema/ischemia (Figure 4) or only supratentorial edema/ischemia. No hemorrhage was detected in the CT scan of the patients. Seven out of 21 patients with putamen involvement (all of whom were identified with bilateral lesions) died in their hospital course. More details of CT findings in the surviving and deceased patients are presented in Table 3.

**Table 3:** Distribution of Brain CT Findings in Methanol Poisoning Patients

**CT findings**		**Total ** No. (%^*^)	**Survived** No. (%^**^)	**Dead** **No. (%)**	***P*** ** Value**
Putamen ischemia/necrosis (n = 21) (%)	Unilateral (n = 5) (%)	5 (10.4)	5(100)	0 (00.0)	0.390
	Bilateral	16 (33.3)	9(56.2)	7(43.8)	0.004
External capsule ischemia (n = 2)	Unilateral	1 (2.1)	0 (00.0)	1(100)	0.138
	Bilateral	1 (2.1)	0 (00.0)	1(100)	
insular subcortex white matter ischemia (n = 4)	Unilateral	1 (2.1)	0 (00.0)	1(100)	0.138
	Bilateral	3 (6.2)	2(66.7)	1(33.3)	
Frontal lobe white matter ischemia (n = 1)	Unilateral	1 (2.1)	1(100)	0 (00.0)	1.00
Occipital lobe white matter ischemia (n = 2)	Unilateral	2 (4.2)	2(100)	0 (00.0)	1.00
Parietal Lobe white matter ischemia (n = 6)	Unilateral	4 (8.3)	3(75)	1(25)	0.278
	bilateral	2 (4.2)	1(50)	1(50)	
Generalized supratentorial edema/ischemia (n = 3)	-	3 (6.2)	2(66.7)	1(33.4)	0.558
Generalized supratentorial and infratentorial edema/ischemia (n = 4)	-	4 (8.3)	0 (00.0)	4(100)	0.044
Thalamus ischemia(n = 3)	Unilateral	1 (2.1)	0 (00.0)	1(100)	-
	bilateral	2 (4.2)	1(50)	1(50)	
Fronto-temporal white matter ischemia(n = 1)	unilateral	1 (2.1)	1(100)	0 (00.0)	1.00
Temporo-parietal white matter ischemia (n = 1)	Unilateral	1 (2.1)	0 (00.0)	1(100)	0.173

^*^Percentage in row (radiological findings). ^**^Percentage in unilateral/bilateral.

###  Data Analysis

 We explored the data obtained from our study population to find probable relationships between clinical, para-clinical, and CT findings of the patients and their outcomes (Tables 4 and 5).

**Table 4:** Crude Measure of Association Between Background Factors and Death or LOS

**Factor**	**Association With Death**^a^	**Association With LOS**^b^
Gender (ref.: female)		
Male	1.21 (0.31, 4.70)	-0.94 (-4.32, 2.43)
Nausea	0.27 (0.11, 0.74)	-1.96 (-4.43, 0.51)
Vomiting	0.40 (0.15, 1.07)	-1.36 (-3.78, 1.07)
No blurred vision	4.31 (1.57-11.85)	0.73 (-1.93, 3.39)
Headache	0.17 (0.02, 1.36)	-0.66 (-3.32, 2.00)
Vertigo	0.18 (0.02, 1.44)	-1.42 (-4.04, 1.21)
LOC	34.62 (8.10, 147.85)	4.41 (2.97, 5.84)
Pulse	1.00 (0.97, 1.03)	0.06 (0.002, 0.113)
Temp	1.97 (0.46, 8.54)	0.93 (-0.54, 2.40)
O_2_ sat	0.91 (0.83, 0.99)	-0.24 (-0.42, -0.06)
HCO_3_	0.68 (0.54, 0.86)	-0.5 (-0.14, 0.04)
PCO_2_	1.03 (1.00, 1.07)	0.01 (-0.05, 0.06)
CPK	1.00 (1.00, 1.00)	0.0017 (0.0012, 0.0022)
LDH	1.00 (1.00, 1.00)	0.005 (0.003, 0.008)
BS (per 10 mg/dL)	1.17 (1.08- 1.27)	0.008 (-0.013, 0.028)
Ca	0.51 (0.23, 1.15)	-0.92 (-2.33, 0.49)
phosphate	0.99 (0.97, 1.02)	0.004 (-0.01, 0.02)
AST	1.01 (1.00, 1.02)	0.02 (0.01, 0.04)
ALT	1.00 (0.99, 1.01)	0.02 (0.004, 0.03)
Normal eye examination	0.65 (0.14, 3.11)	2.67 (1.61, 3.73)
Admission to ICU	27.70 (8.01, 95.73)	3.66 (1.78, 5.53)
Number of days	1.12 (1.00, 1.26)	NA
Abnormal CT	14.05 (3.78- 52.34)	-0.25 (-1.76, 1.26)
Acidosis	1.00 (1.00, 1.00)	4.53 (1.44, 7.62)
SBP (per 10 mm Hg)	0.48 (0.33. 0.69)	0.20 (-0.30, 0.71)
pH (per 1% increase)	0.34 (0.23, 0.52)	-0.07 (-0.11, -0.02)
Age	1.06 (1.01, 1.11)	0.02 (-0.06, 0.10)

LOC, level of consciousness; LOS, length of hospital stay; SBP, Systolic blood pressure; CPK, Creatine phosphokinase; LDH, Lactate dehydrogenase; AST, Aspartate aminotransferase; ALT, Alanine transaminase. ^a^Odds ratio; ^b^Regress coefficient.

**Table 5:** Factors Associated with Different Outcomes of Methanol Poisoning in the COVID-19 Era, Shiraz, Iran

**Variable**	**Crude Effect measure (95% CI)**	**Adjusted**^a^ **Effect measure (95% CI)**
**Outcome: Death**^b^		
Ignoring CT findings and LOC		
No blurred vision	4.31 (1.57-11.85)	18.0 (1.68- 191.9)
pH (per 1% increase)	0.34 (0.23- 0.52)	0.37 (0.22- 0.65)
BS (per 10 mg/dL)	1.17 (1.08- 1.27)	1.13 (1.02- 1.26)
Including CT findings and LOC		
Abnormal CT scan	14.05 (3.78- 52.34)	4.25 (0.91- 19.95)
LOC	34.62 (8.11- 147.85)	20.97 (4.61- 95.42)
Including the LOC without CT findings		
LOC	34.62 (8.11- 147.85)	42.2 (2.35- 756.50)
pH (per 1% increase)	0.34 (0.23- 0.52)	0.45 (0.25- 0.83)
BS (per 10 mg/dL)	1.17 (1.08- 1.27)	1.02 (1.01- 10.3)
**Outcome: LOS**^c^
Including the LOC, ignoring CT findings		
LOC	4.41 (2.97, 5.84)	4.89 (1.98, 7.80)
Pulse	0.06 (0.002, 0.113)	-0.01 (-0.06, 0.04)
ALT	0.02 (0.004, 0.03)	0.01 (-0.0, 0.02)
Including CT findings, ignoring the LOC		
Supratentorial edema	17.55 (16.95-18.16)	-

LOC, Level of consciousness; LOS, length of stay in hospital; BS, blood sugar; ALT, alanine aminotransferase; NS, not significant. ^a^Adjusted for potential confounder factors including no blurred vision, PH, BS, abnormal CT scan, LOC, ALT, supratentorial edema. ^b^Effect measure is odds ratio. ^c^LOS is analyzed for patients who survived up to discharge. Regression coefficients are considered as effect measures.

 According to the results, a significant association was found between nausea on admission and death: 9.9% of patients who presented with nausea died compared to 28.5% of patients without nausea (*P* value = 0.008). No significant difference was observed between patients with and without nausea in the time elapsed from methanol consumption until admission (1.21 vs 1.25; *P *value = 0.758).

 Absence of blurred vision was associated with increased mortality (odds ratio [OR]: 18.0, 95% CI: 1.68 to 191.9).

 All of the hypotensive patients (n = 5) at the time of admission died. A significant association was observed between hypotension and mortality (100.0% vs 0.0%; *P* value < 0.001). Although time from consumption to admission was longer in patients without hypotension compared with hypotensive patients, the difference was not statistically significant (1.24 vs 1 day; *P *value = 0.464)

 Level of consciousness (OR: 42.2; 95% CI: 2.35- 756.50) was associated with death. Patients who were conscious on admission were more likely to survive. There was a significant association between the LOC and poor outcome (*P *value < 0.001).

 Serum phosphate levels had an association with mortality in univariate analysis (*P *value = 0.003). Phosphate levels in patients who survived were about 2 times higher than the deceased patients. However, in multivariate analysis, it was not found to be an independent prognostic factor.

 In our study, elevated BS levels were found to be associated with poor outcome (OR: 1.13, 95% CI: 1.02 to 1.26).

 There was an association between blood pH and increased mortality (OR: 0.37, 95% CI: 0.22 to 0.65). Severe acidosis (pH ≤ 7.2) was documented for 64.2% (n = 79) of patients. Severe acidosis was significantly associated with mortality (*P* < 0.001). No death was observed among patients without severe acidosis.

 There was no significant difference between patients admitted with and without severe acidosis in terms of the time from methanol consumption to admission (1.29 vs 1.14 days; *P* = 0.298).

 Mortality rate in patients with abnormal CT scans was 4 times higher than subjects without abnormality on CT images (OR: 4.25, 95% CI: 0.91 to 19.95).

 Bilateral putamen ischemia/necrosis and generalized supra- and infra-tentorial edema/ischemia were found to have an association with death in this study population (P-value = 0.004 and 0.044 respectively).

 Putamen involvement, especially bilateral putamen involvement, was significantly higher among patients who were admitted with severe acidosis (*P* = 0.016).

 No death was observed among patients with unilateral putamen involvement. All patients with generalized supra- and infra-tentorial edema/ischemia died. Supratentorial edema (RC: 17.55; 95% CI: 16.95- 18.16) were associated with LOS.

###  Other CT Findings Did not Have an Association with Mortality

 The results of the analysis regarding LOS in the hospital showed that patients with severe acidosis had a longer LOS compared with patients without acidosis (3.4 vs 2.2 days; *P* = 0.037). The presence of ischemia/ necrosis in the temporoparietal region and external capsule and also generalized supra-tentorial ischemia/edema were associated with longer LOS (12, 3, 9 days respectively). If CT findings were not taken into account, patients with decreased LOC had longer LOS in the hospital (2 days).

 No statistically significant difference was observed between the two types of treatment regarding the number of deceased and surviving patients (*P* = 0.123, 0.124).

## Discussion

 With the outbreak of COVID-19 in February and early March 2020 in Iran, different ways of protecting against the infection were propagated all over the media including use of vitamins, supplements, herbal medicine, and also alcoholic products. As in any other Islamic country, production, distribution, and use of alcoholic beverages is banned in Iran. At the start of the COVID-19 pandemic, the surge in the consumption of home-made or smuggled alcohol increased, and an outbreak of methanol poisoning took place in March 2020. One of the most affected provinces was Fars.^12^ Therefore, we decided to report the clinical and paraclinical characteristics of methanol poisoning in our population to help in recognizing factors that may have been associated with outcomes in these patients.

 Methanol poisoning is a life-threatening condition that has only been evaluated in few studies. The majority of our population were men which is in line with previous reports showing the predominance of male subjects in methanol poisoning in our country^7,8^ which may result from our cultural issues.

 Gastrointestinal symptoms including nausea (66%) and vomiting (63%) and blurred vision were the most common complaints in our patients, which is comparable to a previous study.^13^ Moreover, we found that patients with nausea and blurred vision had a better outcome with decreased mortality.

 The estimated mean time from methanol consumption to admission at the hospital was 1.5 ± 1.0 days. Presence of nausea was presumed to lead to earlier admission and therefore better prognosis as previously reported^14^; however, no difference in time of hospital admission was observed between patients with or without nausea in this study. Thus, the better prognosis of patients with nausea may not be related to the time of their hospital admission.

 Hypotension at the time of admission was found as a poor prognostic factor in our study population; all patients with hypotension on admission died. Therefore, hypotension and compensatory tachycardia may be considered as alarming signs and their early detection may alter the prognosis of a patient.^15^ In one study,^14^ the authors stated that hypotension in methanol poisoning patients is a result of acidosis. Another study suggested that the formation of formic acid in cardiac tissue as a result of methanol metabolization rather than acidosis may cause myocardial depression^16^; either mechanism may be involved in causing hypotension in such patients and may impact patients’ outcome.

 As stated in previous studies,^14,17^ comatose state on admission was associated with poorer outcome (*P* = 0.00). The result of multivariable analysis showed the association of decreased LOC with the presence of an abnormal CT scan result, increasing mortality rate by 20 times, and also with the presence of acidosis and increased BS levels, increasing mortality rate by 42 times.

 Hypophosphatemia could result in skeletal muscle weakness and rhabdomyolysis, especially following chronic alcohol use and may also lead to the development of metabolic acidosis.^18^ These manifestations may predispose the patient to increased morbidity and mortality. Serum phosphate level was not found to be an independent prognostic factor of mortality but it may be accounted as a contributory factor in this regard. Therefore, correction of phosphate levels can improve outcomes in such patients.

 There is debate on the role of BS levels and hyperglycemia and its variable impact^7-9^ as a prognostic factor in the outcome of patients with methanol poisoning. We found that elevated BS levels are associated with poorer outcomes. Each 1 mg/dL increase in BS could increase one percent probability of death in poisoned patients. The proposed possible mechanisms for hyperglycemia in this situation are stress-induced hyperglycemia and its associated acute pancreatitis. In our patients, there was no clinically suspected acute pancreatitis; therefore, the first mechanism is more probable.

 Severe acidosis was associated with increased mortality among the subjects. Therefore, severe acidosis may also be accounted as a poor prognostic factor in this situation. Gulen et al also reported pH and HCO_3_ levels as significant factors affecting patients’ outcomes.^14^ The mechanism of metabolic acidosis in methanol poisoning could be attributed to formic acid and lactate formation.^19^

 Among all our admitted patients, 65 cases had a normal brain CT scan. This finding is compatible with previous studies that claimed that in the acute phase of methanol poisoning, brain CT may show no abnormality.^10^ On the other hand, mortality rate in patients with abnormal CT scans was 4 times higher than subjects without abnormality on CT images. Hence, regardless of the type of abnormal CT finding, the presence of an abnormal CT scan should be considered as a poor prognostic factor.

 Putaminal ischemia/necrosis was the most prevalent CT scan finding among our patients, which was compatible with the result of previous studies.^10,20^ Several possible mechanisms have been suggested for this phenomenon. According to Fontenot and Pelak, higher accumulation of formic acid in the putamen compared to other zones in the brain is associated with its direct toxic effects on putamen.^21^ Also, due to the higher sensitivity of putamen, this area of the brain is expected to be more affected by acid accumulation.^21^ The relationship between radiological findings and patients’ outcomes has long been a matter of debate.^10,22^ A direct relationship between bilateral putaminal ischemia/necrosis and generalized supra- and infra-tentorial edema/ischemia with mortality rate was observed but this finding was not proven to be an independent prognostic factor in multivariate analysis. Putamen involvement, especially bilateral putamen involvement, was significantly higher among patients with severe acidosis. Indeed, the relation of putaminal involvement with death may be secondary to severe acidosis. In our study population, there were no deceased cases among patients with unilateral putaminal necrosis; therefore, there was no relation between this finding and death.

 Four of the patients had generalized supra- and infra-tentorial edema/ischemia in their CT scan and all 4 patients died.

 According to the results of this study, we could postulate that bilateral putamen involvement and generalized supra- and infra-tentorial edema/ischemia could be alarming radiologic findings in methanol poisoning. In contrast to a previous study,^10^ we did not find a significant association between insular subcortical white matter necrosis and the patients’ mortality rate.

 The present study showed an association between increased LOS with severe acidosis, decreased LOC, and CT manifestations including generalized supra-tentorial ischemia/edema. Hence, longer LOS is expected in patients carrying poor prognostic signs.

 Different therapies including gastric lavage, alcohol dehydrogenase enzyme blockade with the use of fomepizole or ethanol, dialysis, alkalization, and treatment with folate have been used in treating methanol poisoning patients.^14,23^ Due to the unavailability and high costs of fomepizole, ethanol was used as its alternative to block the formation of the toxic metabolite. In this study, the impact of management with either hemodialysis or oral ethanol on patients’ outcomes was evaluated. We did not find any statistically significant relationship between mortality rates and the use of either treatment.

 Our study had some limitations. Confirmation of methanol poisoning based on clinical features and non-specific lab data rather than measuring the exact methanol metabolite in the blood, due to the unavailability of standardized chromatographic methods at our institutions, are among limitation that must be mentioned for this study. Moreover, due to the sample size, some findings in some subcategories were not very robust. Nevertheless, this study was one of the largest cohorts regarding methanol poisoning.

## Conclusion

 Among clinical findings, nausea and blurred vision were associated with a better prognosis in methanol poisoning. Hypotension, decreased LOC, severe acidosis, and increased BS levels were found to be poor prognostic factors. In the acute phase of methanol poisoning, brain CT may show no abnormality. Regardless of the type of abnormal CT finding, mortality rate in patients with abnormal CT scans was 4 times higher than subjects without abnormality on CT images. Among CT manifestations, bilateral putaminal ischemia/necrosis and generalized supra- and infra-tentorial edema/ischemia should be considered as alarming radiologic findings in methanol poisoning. Patients with severe acidosis, decreased LOC and CT manifestations may have longer LOS in the hospital.
